# Intraovarian Platelet-Rich Plasma Therapy for PCOS: Unanswered Questions and Future Research Directions

**DOI:** 10.3390/jcm14248845

**Published:** 2025-12-14

**Authors:** Zaher Merhi

**Affiliations:** 1 Reproductive Endocrinology and Infertility, Rejuvenating Fertility Center, New York, NY 10019, USA; zaher.merhi@rfcfertility.com; Tel.: +1-(203)-557-9696; 2 Department of Obstetrics and Gynecology, Division of Reproductive Endocrinology and Infertility, Maimonides Medical Center, Brooklyn, NY 11219, USA; 3 Department of Obstetrics and Gynecology, Division of Reproductive Endocrinology and Infertility, Albert Einstein College of Medicine, Bronx, NY 10461, USA

**Keywords:** PRP, PCOS, ovaries, ovulation, pregnancy

## Abstract

**Background:** Polycystic ovary syndrome (PCOS)-related infertility remains a major challenge and the efficacy of conventional treatments is limited in certain patient groups and often fails to address the underlying causes of ovarian dysfunction. Platelet-rich plasma (PRP) is rich in growth factors and cytokines and has emerged as a potential regenerative therapy for women with a diminished ovarian reserve. **Methods:** A literature search for studies pertaining to intraovarian PRP administration and PCOS was performed on PubMed. **Results:** Preclinical studies in PCOS animal models have demonstrated that intraovarian PRP can improve folliculogenesis, enhance antioxidant defenses, normalize steroid hormone levels, and downregulate pro-apoptotic pathways. Early clinical reports suggest that intraovarian PRP may restore ovulation and improve ovarian reserve in women with long-standing amenorrhea and poor responses to standard fertility treatments. The proposed mechanisms of how PRP could improve folliculogenesis include the modulation of local ovarian gene expression, the activation of dormant follicles, angiogenesis, and a reduction in oxidative stress and inflammation. **Conclusions:** Although preliminary data are promising, larger studies are needed to establish the efficacy, if any, of intraovarian PRP administration as a potential novel therapeutic adjunct in women with PCOS.

## 1. Introduction

Polycystic ovary syndrome (PCOS) is a leading cause of infertility in reproductive-aged women, primarily due to chronic anovulation and disrupted follicular development. The condition is marked by elevated androgen levels, irregular or absent ovulation, and polycystic ovarian morphology. These reproductive abnormalities are often compounded by metabolic issues, such as insulin resistance and obesity, that further impair ovulatory function and fertility outcomes [1]. In PCOS, disruptions in gonadotropin signaling, most notably elevated pituitary luteinizing hormone (LH) levels, along with the reduced production of estradiol (E2) and progesterone (P4) and abnormal gene expression of the two estrogen receptors ERα and Erβ, play a role in impairing normal folliculogenesis [2,3,4,5]. These hormonal imbalances are compounded by an overexpression of the pro-apoptotic transcription factor c-Myc, which accelerates granulosa cell death and promotes follicular atresia, ultimately contributing to infertility [2,3,4,5]. Platelet-rich plasma (PRP) is an experimental fertility treatment that uses a patient’s own plasma and platelets, rich in growth factors, to potentially improve oocyte quality, enhance ovarian function, and thicken the endometrial lining [6,7,8,9]. It has been used for women with a low ovarian reserve and premature ovarian insufficiency, as well as for recurrent implantation failure in in vitro fertilization (IVF), with injections administered directly into the ovaries or uterus to stimulate cellular regeneration and receptivity [6,7,8,9]. Because PRP is rich in biologically active growth factors, such as Transforming Growth Factor-β (TGF-β), Vascular Endothelial Growth Factor (VEGF), Platelet-Derived Growth Factor (PDGF), and Insulin-like Growth Factor (IGF) [10], and has demonstrated regenerative and anti-apoptotic effects in various tissues [11], several recent studies have investigated its potential therapeutic role in PCOS.

## 2. Methods

A review of the literature for peer-reviewed basic science, animal, and human studies was performed in PubMed. The search used the following keywords: “Platelet rich plasma and PCOS” and “PRP and PCOS”. Inclusion criteria included PRP administration directly into the ovaries, PCOS animal models, and women with PCOS. Studies related to ovarian dysfunction due to conditions other than PCOS were excluded. The AI tool ChatGPT 5.2 was used only for language editing and formatting only.

## 3. Results

In 2019, an experimental animal study by Seyyed Anvari et al. [12] studied the effect of PRP administration on PCOS-induced ovarian dysfunction. In that study, a PCOS model was produced by giving animals daily injections of dehydroepiandrosterone (DHEA) for a total of 15 days. A total of 30 immature Sprague Dawley female rats (21 days old) were randomized into five groups (*n* = 6/group): a control group (30 days, no treatment), PCOS-induced group sampled after 15 days, PCOS-induced group sampled after 30 days, PCOS-induced group that received autologous PRP sampled after 15 days, and PCOS-induced group that received autologous PRP sampled after 30 days. The PRP was extracted, activated by adding calcium, and then injected into the ovarian mesovarium. Outcomes included serum hormone levels (E2, P4, testosterone [T], androstenedione, follicle-stimulating hormone [FSH], and LH), oxidative stress markers (total antioxidant capacity [TAC], malondialdehyde [MDA], superoxide dismutase [SOD], glutathione peroxidase [GSH]), histology (preantral, antral, and atretic follicle counts, corpora lutea formation), molecular markers (ERα, ERβ, c-Myc expression measured using RT-PCR and immunohistochemistry), and RNA integrity (acridine orange staining). Their results demonstrated that, compared with untreated PCOS animals, PRP-treated rats showed a decreased RNA damage and a marked increase in intact follicles (both preantral and antral), as well as a marked increase in corpora lutea formation, indicating more ovulatory events. PRP significantly upregulated ERα and ERβ expression, while suppressing c-Myc, thus mitigating apoptosis and supporting follicular development. Antioxidant assays revealed that PRP restored redox balance by increasing TAC, SOD, and GSH and reducing MDA levels. Endocrine profiles also improved: while PCOS animals exhibited elevated FSH, LH, T, and androstenedione, with suppressed E2 and P4, PRP-treated groups demonstrated normal androgens and gonadotropins alongside a restitution of E2 and P4 synthesis. Their findings suggested that PRP exerts effects at multiple levels—including endocrine regulation, antioxidant enhancement, anti-apoptotic modulation, and pro-folliculogenic activity—that together improve ovarian function in PCOS. However, the study was limited by its small sample size, short duration (30 days maximum), focus on molecular and histological markers without functional fertility outcomes, and use of an animal model, thereby necessitating further research in human subjects with longer follow-ups and broader endpoints.

Current pharmacologic treatments for PCOS, including metformin for insulin resistance and clomiphene citrate or letrozole for ovulation induction, often demonstrate a limited effectiveness and may be associated with undesirable side effects [13,14], underscoring the need for regenerative approaches that target both ovarian dysfunction and the underlying metabolic disturbances. PRP and stem cells such as placenta-derived mesenchymal stem cells (PDMSCs) hold strong regenerative properties, as well as anti-inflammatory and angiogenic actions [15]. Both PRP and PDMSCs have demonstrated beneficial effects on ovarian physiology [16,17]. A recent study investigated their individual and combined roles in a letrozole-induced rat PCOS model [18]. In that study, 25 adult Wistar rats were assigned to five groups (*n* = 5 per group): sham (given 1 dose of 1 mL of carboxymethylcellulose solution), PCOS-induced by letrozole 1 mg/kg, PDMSCs (single intraovarian dose), PRP (single intraovarian dose), and PDMSCs + PRP as a single intraovarian dose. PDMSCs were extracted from human placentas and characterized by the markers (CD34^−^, CD45^−^, CD73^+^, CD90^+^, and CD105^+^), while PRP was isolated from the rat plasma and activated with calcium and thrombin. Each treatment consisted of a single intraovarian injection, and outcomes were evaluated 14 days later using histological analysis, hormone profiling, metabolic assessments, and inflammatory markers. The PCOS group exhibited numerous cystic follicles, reduced healthy follicles, absent corpora lutea, elevated T, LH, fasting glucose, and insulin blood levels, homeostasis model assessment of insulin resistance (HOMA-IR), tumor necrosis factor-alpha (TNF-α), and interleukin-6 (IL-6), along with reduced E2 and FSH. Both PDMSCs and PRP alone partially reversed these abnormalities, but the combined therapy had a synergistic effect, significantly increasing primordial, primary, secondary, and antral follicles and corpus lutea while decreasing cystic follicles, lowering testosterone and LH, restoring FSH and E2, reducing fasting blood glucose, fasting insulin, and HOMA-IR, and markedly decreasing pro-inflammatory cytokines TNF-α and IL-6. Mechanistically, these effects may be explained by the anti-apoptotic, angiogenic, and paracrine actions of PDMSCs and PRP, including the activation of the PI3K/AKT and TGF-β signaling pathways, improved angiogenesis, inhibition of granulosa cell apoptosis, modulation of steroidogenic enzymes, enhanced glucose transporter type 4 (GLUT4), and suppression of the proinflammatory NF-κB. Despite limitations such as the short follow-up period and use of an animal model without fertility endpoints, the findings indicate that PDMSCs and PRP, especially when used together, may offer a promising regenerative strategy for PCOS by concurrently improving ovarian function, metabolic parameters, and inflammatory pathways. These results support the need for further translational research and well-designed clinical trials in humans.

At the time of writing this manuscript, there were only two case reports in humans that evaluated the impact of PRP intraovarian administration in women with PCOS. It is known that PCOS is often accompanied by poor oocyte quality, which limits the success of assisted reproductive technologies (ARTs) [19,20]. In one case report [21], a 34-year-old woman with PCOS and a diminished ovarian reserve (antral follicle count [AFC] 0 in the right ovary and 2–3 in the left; FSH 10.99 IU/mL) presented with four years of primary infertility. Her 35-year-old partner had hypertension and severely abnormal sperm morphology (96%). The couple had previously undergone two unsuccessful intrauterine inseminations (IUIs) and one failed IVF–ICSI cycle (immature and degenerated oocytes were produced). After six months, the patient received bilateral intraovarian PRP treatment, prepared from 10 to 12 mL of autologous blood using a double-centrifugation protocol and injected under ultrasound guidance. Following PRP, the patient’s AFC increased to 12, and subsequent ovarian stimulation produced seven good-quality oocytes, resulting in several embryos. Her fresh embryo transfer was unsuccessful, but a subsequent frozen embryo transfer achieved a pregnancy. These findings align with previous studies reporting that intraovarian PRP improves AFC, oocyte yield, and pregnancy outcomes in poor ovarian responders, likely through growth factors that promote angiogenesis, follicle activation, and oocyte competence. Although this case showed that intraovarian PRP could improve ovarian function and enhance ART outcomes in PCOS with diminished ovarian reserves, its interpretation is limited by the single-patient design, absence of a control group, and variability in PRP preparation methods. Nonetheless, it supports the growing evidence that PRP may serve as a regenerative adjunct in infertility treatment, warranting validation in larger randomized controlled trials to establish efficacy, safety, and long-term reproductive outcomes.

Another case report described the first instance of a woman with long-standing amenorrhea due to PCOS who had been attempting to conceive and subsequently regained spontaneous ovulatory cycles, along with notable improvements in multiple hormonal imbalances, following intraovarian PRP treatment [22]. The patient was a 35-year-old obese woman with PCOS (BMI 39 kg/m^2^), type 2 diabetes, hypertension, and hyperandrogenism who presented with long-term amenorrhea and infertility. On pelvic ultrasound, she had enlarged PCOS-looking ovaries (AFC = 15 in each ovary), an elevated testosterone of 140.3 ng/dL, elevated 17-hydroxyprogesterone of 218 ng/dL, fasting glucose of 142 mg/dL, and a significantly increased C-reactive protein (CRP) level (44.4 mg/L). Approximately 40 mL of autologous blood was processed to obtain 4 mL of PRP, which was injected into the ovarian cortex using a 22-gauge needle (2 mL/ovary; 5–7 punctures/ovary). Within 10 days, the patient developed a spontaneous dominant follicle (14.5 mm) accompanied by a rise in estradiol (from 80.9 to 208 pg/mL), followed by ovulation, confirmed by an increase in progesterone (3.05 ng/mL) and visualization of a corpus luteum. Menstrual cycles resumed two weeks later, and both testosterone (dropped from 140.3 to 20.8 ng/dL) and CRP showed a substantial improvement. During the following cycle, a new dominant follicle reached 18 mm with normal estradiol and LH dynamics, and intrauterine insemination was performed, although pregnancy was not achieved. Overall, intraovarian PRP led to spontaneous ovulation, the normalization of androgen levels, improved follicular development, and the restoration of menses for up to three months. While spontaneous ovulation unrelated to PRP, or a mechanical effect similar to ovarian drilling, cannot be entirely excluded, the accompanying hormonal and inflammatory improvements suggest a genuine biological response to PRP components. This case underscores the potential of PRP to restore ovulatory and endocrine dysfunction in PCOS; however, larger controlled studies with long-term reproductive and metabolic follow-up are essential before considering routine clinical application.

**Figure 1:**
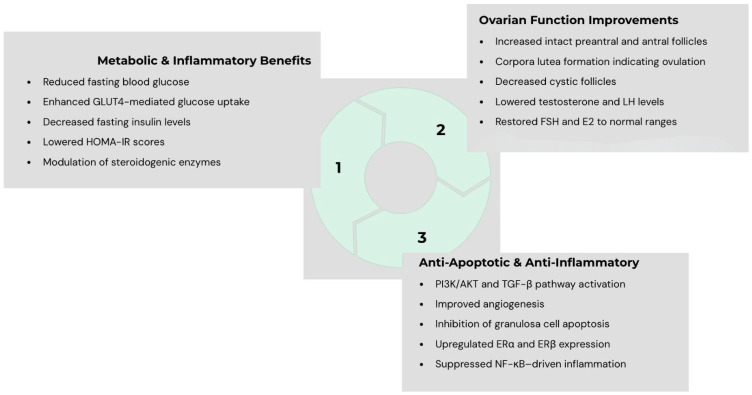
Proposed mechanisms through which platelet-rich plasma (PRP) may exert multi-level beneficial effects in polycystic ovary syndrome (PCOS), including endocrine regulation, enhancement of antioxidant defense, modulation of apoptosis, and stimulation of folliculogenesis—collectively contributing to improved ovarian function in PCOS.

Table 1 summarizes the studies and their findings. Figure 1 presents a summary of possible mechanisms by which PRP could exert its effects on PCOS states.

## 4. Discussion

The therapeutic potential of PRP in PCOS could lie in its ability to act directly on the ovarian microenvironment, which is disrupted by chronic hyperandrogenism, oxidative stress, and altered folliculogenesis (see Figure 1). Animal studies have demonstrated that intraovarian PRP could restore hormonal balance. At the molecular level, PRP has been shown to restore the expression of genes pertinent to ovulation and folliculogenesis. These effects translate into improved follicular survival, reduced atresia, and enhanced corpus luteum formation in animal models. Emerging human data, though limited to small case reports, align with these findings.

Significant limitations exist despite these encouraging observations so far. The current evidence is derived mainly from animal models focusing on molecular and histological markers without functional fertility and pregnancy outcomes, human case reports, and short-term follow-up periods. The mechanisms underlying PRP’s effects remain incompletely defined, with proposed pathways including angiogenesis, immune modulation, improved granulosa cell function, and the activation of dormant follicles via paracrine signaling. Importantly, the long-term safety profile of intraovarian PRP—particularly in a population already at an increased risk of metabolic alterations—requires rigorous evaluation. One significant limitation in clinical practice is the heterogeneous protocols for PRP preparation and administration. For instance, things that vary significantly among clinics worldwide include the method of PRP preparation (so many various kits are now available commercially), the size/gauge of the needles used to inject PRP into the ovaries, the injected areas within the ovaries (i.e., cortical versus medullary), and the number of PRP injections per ovary. Therefore, the need for a standardized protocol for clinicians is evident.

## 5. Conclusions

Future research should prioritize larger human cohort studies and, ideally, randomized controlled trials that use standardized protocols for PRP preparation, dosing, and administration in women with PCOS. Key outcomes should include ovulation rates, live birth rates, metabolic improvements, and safety endpoints. Furthermore, mechanistic studies are warranted to delineate the exact molecular pathways through which PRP influences folliculogenesis and steroidogenesis in PCOS. If validated, PRP could represent a paradigm shift in the management of PCOS-related infertility, providing a minimally invasive, autologous, and potentially regenerative alternative for women resistant to conventional therapies.

## Figures and Tables

**Table 1:** Summary of PRP in PCOS: animal studies and human case reports.

Study Type	Hormonal Findings Following Intraovarian PRP	Ovarian Gene Expression/Pathway Findings	Ovarian/Clinical Outcomes
Animal Study (Seyyed Anvari et al. [12])	↓ LH, ↓ FSH, ↓ T, ↓ A; ↑ E2, ↑ P4	↑ ERα, ↑ ERβ; ↓ c-Myc; ↑ SOD, ↑ GSH-px; ↓ MDA	↑ Healthy follicles, ↑ Corpora lutea; ↓ Cystic/atretic follicles; ↓ RNA damage
Animal Study (Sarvestani et al. [18])	↓ T, ↓ LH; ↑ E2, ↑ FSH	↑ PI3K/AKT, ↑ TGF-β, ↑ GLUT4; ↓ NF-κB	↑ Primordial, primary, secondary, antral follicles; ↑ Corpus luteum; ↓ Cystic follicles
Human Case Report#1 (34 years old, low ovarian reserve)	*Baseline:* AMH = 1.45 ng/mL, AFC 0–3, FSH 10.99 IU/mL *Post-PRP:* AFC ↑ to 12, 5 MII oocytes	Not assessed in this study	Improved ovarian reserve, multiple embryos, pregnancy after frozen embryo transfer
Human Case Report#2 (35 years old, long-term amenorrhea)	*Baseline:* T = 140.3 ng/dL, CRP = 44.4 mg/L *Post-PRP:* T ↓ to 20.8 ng/dL, ovulation resumed	Not assessed in this study	Spontaneous ovulation, cycles resumed, improved hormonal balance

Abbreviations: LH = luteinizing hormone; FSH = follicle-stimulating hormone; T = testosterone; A = androstenedione; E2 = estradiol; P4 = progesterone; ER = estrogen receptor; SOD = superoxide dismutase; GSH-px = glutathione peroxidase; MDA = malondialdehyde; PI3K/AKT = phosphoinositide 3-kinase/protein kinase B pathway; TGF-β = transforming growth factor-beta; GLUT4 = glucose transporter type 4; NF-κB = nuclear factor kappa-light-chain-enhancer of activated B cells; AMH = anti-Müllerian hormone; AFC = antral follicle count; CRP = C-reactive protein; MII = metaphase II oocyte; PRP = platelet-rich plasma; ↑ = increased or upregulated; ↓ = decreased or downregulated.

## Data Availability

No new data were created or analyzed in this study.
